# Influence of overdistension/recruitment induced by high positive end-expiratory pressure on ventilation–perfusion matching assessed by electrical impedance tomography with saline bolus

**DOI:** 10.1186/s13054-020-03301-x

**Published:** 2020-09-29

**Authors:** Huaiwu He, Yi Chi, Yun Long, Siyi Yuan, Inéz Frerichs, Knut Möller, Feng Fu, Zhanqi Zhao

**Affiliations:** 1 Department of Critical Care Medicine, Peking Union Medical College Hospital, Peking Union Medical College, Chinese Academy of Medical Sciences, Beijing, China; 2 Department of Anesthesiology and Intensive Care Medicine, University Medical Center of Schleswig-Holstein Campus kiel, Kiel 24105, Germany; 3 Institute of Technical Medicine, Furtwangen University, Villingen-Schwenningen, Germany; 4 Department of Biomedical Engineering, Fourth Military Medical University, 169 Changle Xi Rd, Xi’an, China

**Keywords:** Electrical impedance tomography, Shunt, Dead space, Ventilation–perfusion matching, Overdistension, Recruitment, Ventilation distribution, Lung perfusion

## Abstract

**Background:**

High positive end-expiratory pressures (PEEP) may induce overdistension/recruitment and affect ventilation–perfusion matching (VQMatch) in mechanically ventilated patients. This study aimed to investigate the association between PEEP-induced lung overdistension/recruitment and VQMatch by electrical impedance tomography (EIT).

**Methods:**

The study was conducted prospectively on 30 adult mechanically ventilated patients: 18/30 with ARDS and 12/30 with high risk for ARDS. EIT measurements were performed at zero end-expiratory pressures (ZEEP) and subsequently at high (12–15 cmH_2_O) PEEP. The number of overdistended pixels over the number of recruited pixels (O/R ratio) was calculated, and the patients were divided into low O/R (O/R ratio < 15%) and high O/R groups (O/R ratio ≥ 15%). The global inhomogeneity (GI) index was calculated to evaluate the ventilation distribution. Lung perfusion image was calculated from the EIT impedance–time curves caused by 10 ml 10% NaCl injection during a respiratory pause (> 8 s). DeadSpace_%_, Shunt_%_, and VQMatch_%_ were calculated based on lung EIT perfusion and ventilation images.

**Results:**

Increasing PEEP resulted in recruitment mainly in dorsal regions and overdistension mainly in ventral regions. ΔVQMatch_%_ (VQMatch_%_ at high PEEP minus that at ZEEP) was significantly correlated with recruited pixels (*r* = 0.468, *P* = 0.009), overdistended pixels (*r* = − 0.666, *P* < 0.001), O/R ratio (*r* = − 0.686, *P* < 0.001), and ΔSpO_2_ (*r* = 0.440, *P* = 0.015). Patients in the low O/R ratio group (14/30) had significantly higher Shunt_%_ and lower VQMatch_%_ than those in the high O/R ratio group (16/30) at ZEEP but not at high PEEP. Comparable DeadSpace_%_ was found in both groups. A high PEEP caused a significant improvement of VQMatch_%_, DeadSpace_%_, Shunt_%_, and GI in the low O/R ratio group, but not in the high O/R ratio group. Using O/R ratio of 15% resulted in a sensitivity of 81% and a specificity of 100% for an increase of VQMatch_%_ > 20% in response to high PEEP.

**Conclusions:**

Change of ventilation–perfusion matching was associated with regional overdistention and recruitment induced by PEEP. A low O/R ratio induced by high PEEP might indicate a more homogeneous ventilation and improvement of VQMatch.

**Trial registration:**

ClinicalTrials.gov, NCT04081155. Registered on 9 September 2019—retrospectively registered.

## Key messages

- The change of ventilation–perfusion matching was associated with regional overdistention and recruitment induced by PEEP.
- A low O/R ratio induced by high PEEP might indicate a more homogeneous ventilation and improvement of VQMatch.
- EIT is capable of measuring regional lung ventilation and perfusion by means of detecting regional ventilation-related gas volume changes and regional blood flow by saline bolus injection, respectively.

## Introduction

In the context of lung protective ventilation, positive end-expiratory pressure (PEEP) is applied to open collapsed lung regions and keep the lung open. PEEP setting is often based on the response of oxygenation and/or respiratory compliance in patients with acute respiratory distress syndrome (ARDS) under mechanical ventilation. Change of oxygenation, as a complex indicator of ventilation–perfusion matching, cannot directly reflect the change of shunt/dead space during the increase of PEEP. In fact, only a weak to moderate correlation was found between oxygenation and lung aeration during the PEEP changes [1, 2]. On the other hand, lung mechanics reflects merely ventilation and not perfusion. A recent clinical study found that PEEP selection based on best global respiratory compliance might result in poor outcomes in the ARDS patients [3]. Because of the high degree of inhomogeneity in the respiratory system of ARDS patients, an increase of PEEP introduces regional lung overdistension and recruitment at the same time. This may subsequently alter shunt and dead space.

Little is known on how the regional lung overdistension and recruitment influence regional ventilation–perfusion (V-Q) matching (shunt and dead space) in response to PEEP increase. Karbing et al. recently found that improved lung aeration following an increase in PEEP and detected by CT scan was not always consistent with reduced shunt and V-Q mismatch by model-based method [4]. It remains a great challenge to assess the effect of PEEP on regional overdistension/recruitment and V-Q matching directly at the bedside.

Electrical impedance tomography (EIT) is a non-invasive, non-radiation, and real-time monitoring method to monitor regional ventilation distribution at the bedside [5]. In recent advance, EIT was proposed to assess regional lung perfusion with saline bolus injection in ICU patients [6–9]. Hence, EIT would be the ideal bedside tool to evaluate the influence of PEEP on V-Q matching. We hypothesized that EIT may help to better explain the relationship between regional lung overdistension and recruitment, shunt and dead space, and V-Q matching during a PEEP increase.

The aim of the study was to investigate the association between lung overdistension/recruitment induced by PEEP and ventilation–perfusion matching in patients suffering from or being at high risk of developing ARDS. Further, we explored a potential novel indicator of regional overdistension/recruitment ratio to assess the response of V-Q matching after PEEP increase.

## Materials and methods

### Study population

The study was approved by the Institutional Research and Ethics Committee of the Peking Union Medical College Hospital. Informed consent was obtained from all patients or next of kin before data were included into the study. The clinical trial registration number was NCT04081155.

When the research team was available from Jan 2019 to May 2020, patients with ARDS or with high-risk ARDS admitted to the Department of Critical Care Medicine of Peking Union Medical College Hospital, who received mechanical ventilation, were screened for eligibility. Diagnosis of ARDS was based on the Berlin definition [10]. High-risk ARDS was defined as those mechanically ventilated patients who had some high risk factors of ARDS (major operation, massive transfusion and trauma, etc.) and lung collapse in the dependent region but with PaO_2_/FiO_2_ > 300 mmHg at the enrollment.

Included patients should have been deeply sedated and a central venous catheter placed for treatment as per clinical decision at the time of enrollment. Patients were excluded from the study in the presence of age < 18 years, pregnancy, ribcage malformation, baseline PEEP > 12 cmH_2_O and SpO_2_ < 88%, and any contraindication to the use of EIT (e.g., automatic implantable cardioverter defibrillator, and implantable pumps).

### Study protocol

Patient demographics and relevant clinical data were collected at the enrollment day, including age, sex, Acute Physiology and Chronic Health Evaluation II score (APACHE II), heart rate, mean arterial pressure, FiO_2_, SpO_2,_ and as outcome the 28-day mortality.

Patients were ventilated under pressure control mode. Ventilator settings were tidal volume 6–8 ml/kg of ideal body weight. PEEP, FiO_2_ was set to maintain SpO_2_ > 90%, and respiratory rate was set to obtain arterial pH of 7.30–7.45 based on the ARDS-Net suggestions [11]. All patients were deeply sedated (Richmond Agitation-Sedation Scale at − 4) and kept in the supine position. The following PEEP adjustment was performed for each patient:

1. PEEP was switched to a zero end-expiratory pressure (ZEEP) for 10 min, and FiO_2_ was titrated to obtain peripheral oxygen saturation (SpO_2_) > 90%.
2. PEEP was increased to a high PEEP level (preferably 15 cmH_2_O) for another 10 min within a single step. If the patient was not able to tolerate 15 cmH_2_O as assessed by the physician (e.g., due to impaired circulation), PEEP of 12 cmH_2_O was used instead.

### Regional ventilation and perfusion measured by EIT

EIT measurements were performed with PulmoVista 500 (Dräger Medical, Lübeck, Germany) throughout the PEEP adjustment. A silicone EIT belt with 16 surface electrodes was placed around the patient’s thorax at the 4th intercostal space level. All patients received standard care. EIT measurements were continuously recorded at 20 Hz. At the end of each PEEP level (i.e., ZEEP and high PEEP), a bolus of 10 ml 10% NaCl was injected during a respiratory pause (at least for 8 s) through the central venous catheter. The EIT data were digitally filtered using a low-pass filter with a cut-off frequency of 0.67 Hz to eliminate periodic cardiac-related impedance changes (for evaluation of both ventilation and perfusion). Perfusion evaluated via saline bolus injection corresponded to non-periodic impedance drop that was not influenced by the low-pass filtering. Further, the data were analyzed offline using customized software programmed with MATLAB R2015 (the MathWorks Inc., Natick, MA).

Ventilation map was equally divided into two non-overlapping horizontal anterior-to-posterior regions of interest, which were denoted as the ventral and dorsal regions. Regional ventilation map was calculated by subtracting the end-expiration from the end-inspiration image, which represents the variation during tidal breathing. The tidal images before the apnea period (2-min period) were averaged to increase the signal-to-noise ratio.

1 $$ {V}_i=\frac{1}{N}{\sum}_{n=1}^N\left(\varDelta {Z}_{i, Ins,n}-\varDelta {Z}_{i, Exp,n}\right) $$

where *V*_*i*_ is the pixel *i* in the ventilation image, *N* is the number of breaths within the analyzed period, and Δ*Z*_*i,Ins*_ and Δ*Z*_*i,Exp*_ are the pixel values in the raw EIT image at the end-inspiration and end-expiration, respectively.

The ventilation gain and loss via PEEP increase were assessed as follows:

2 $$ {\Delta  V}_i={V}_{i\_\mathrm{PEEP}}-{V}_{i\_\mathrm{ZEEP}} $$

Δ*V*_*i*_ > 0 is associated with ventilation gain whereas Δ*V*_*i*_ < 0 with ventilation loss. To improve signal-to-noise ratio, we defined a threshold of 20% of maximum *V*_*i*_. Recruited pixels were defined as pixels *r* that exhibited ventilation gains higher than the threshold:

3 $$ {\Delta  V}_r>20\%\times \max \left({V}_i\right),i\in \left[1,1024\right] $$

Similarly, overdistended pixels were defined as pixels *o* with ventilation loss higher than the threshold:

4 $$ {\Delta  V}_o<-20\%\times \max \left({V}_i\right),\kern1.50em i\in \left[1,1024\right] $$

The number of overdistended pixels over the number of recruited pixels (O/R ratio) was subsequently calculated. With the O/R ratio, we tried to summarize the degrees of overdistension and recruitment with one single index. The patients were divided into low O/R (O/R ratio < 15%) and high O/R groups (O/R ratio > 15%).

Changes of end-expiratory lung impedance (ΔEELI) were determined relative to the reference time point during device calibration. The global inhomogeneity (GI) index [12] was calculated offline.

Due to its high conductivity, 10% NaCl acts as an EIT contrast agent, passes through the pulmonary circulation thereby producing a dilution curve after bolus injection during the apnea period based on the first pass kinetics theory [13, 14]. Regional perfusion map was calculated as the slope of regional impedance–time curves after saline bolus injection [15, 16]. The detailed calculation was described in previous studies [6, 7]. In brief, the regional impedance–time curves during the descending phase were fitted with linear regression:

5 $$ \varDelta {Z}_i(t)={a}_it+b $$

where *t* is the time starting from one cardiac cycle after the initial descent in the global impedance curve caused by saline injection, and ending at the trough of the global curve during the apnea period. *P*_*i*_, the perfusion value of pixel *i* in the perfusion image, was equaled to –*a*_*i*_.

Further, ventilated and perfused regions were defined as follows: Region *k* is ventilated if:

6 $$ {V}_k>20\%\times \max \left({V}_K\right),\kern1.50em K\in \left[1,1024\right] $$

Similarly, region *g* was perfused if:

7 $$ {P}_g>20\%\times \max \left({P}_G\right),\kern1.50em G\in \left[1,1024\right] $$

Subsequently, the following three regions were identified: the area that was only ventilated (*A*_*V*_), the area that was only perfused (*A*_*P*_), and the area that was both ventilated and perfused (*A*_*V+P*_). To correlate with clinical events, the following EIT-derived parameters were calculated according to their physiological definitions:

8 $$ \mathrm{DeadSpac}{\mathrm{e}}_{\%}={A}_V/\left({A}_V+{A}_P+{A}_{V+P}\right)\times 100\% $$

9 $$ \mathrm{Shun}{\mathrm{t}}_{\%}={A}_P/\left({A}_V+{A}_P+{A}_{V+P}\right)\times 100\% $$

10 $$ \mathrm{VQMatc}{\mathrm{h}}_{\%}={A}_{V+P}/\left({A}_V+{A}_P+{A}_{V+P}\right)\times 100\% $$

**Fig. 1:**
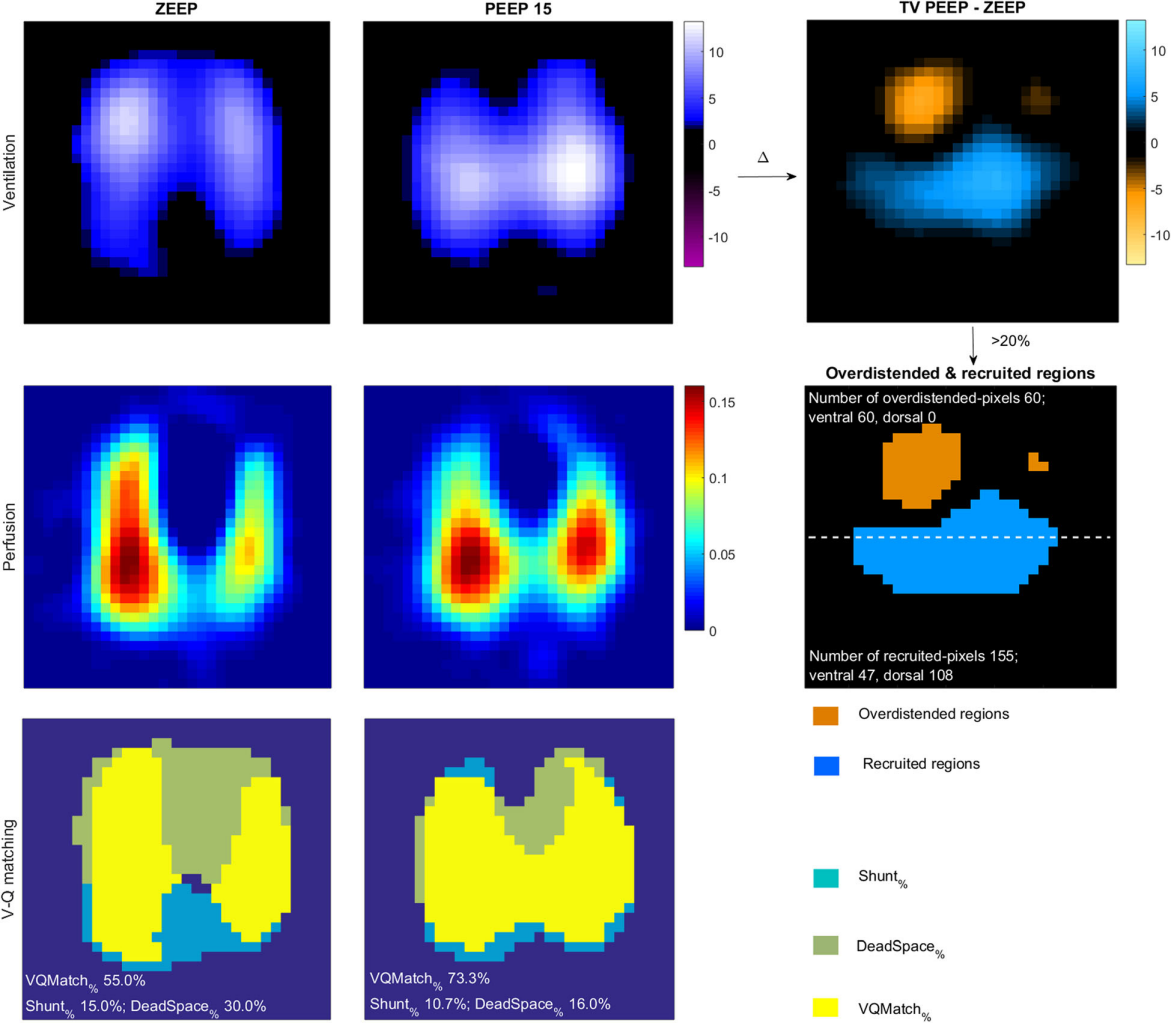
Illustration of the data analysis method of regional recruitment, overdistension, and V-Q matching in one study patient. Top: tidal variation images at ZEEP (left), PEEP (middle), and the corresponding difference image (right). In the tidal variation images, regions with low ventilation are marked in dark blue and highly ventilated regions in light blue to white. Collapsed or overdistended regions are comprised in the low/non-ventilated dark blue or black areas. In the difference image, ventilation gain and loss at PEEP compared to ZEEP are marked in blue and orange, respectively. Middle: perfusion images at ZEEP (left) and PEEP (middle). Highly perfused regions are marked in red. Colorbars in arbitrary units. Recruited and overdistended regions were defined based on ventilation difference image (right). Bottom: regional V-Q matching images at ZEEP (left) and PEEP (right)

Figure 1 illustrates the analysis with patient data. The tidal impedance variation during normal tidal breathing before apnea was used for the calculation of ventilation-related parameters. The impedance–time curve caused by saline bolus during the apnea period was used for the perfusion-related parameters. The regional recruited and overdistended pixel distribution image was derived from the difference of ZEEP and high PEEP ventilation tidal images (Fig. 1 top). The regional V-Q images (Fig. 1 bottom) were derived from the difference of ventilation and perfusion images at the same PEEP level.

### Statistical analysis

A descriptive analysis was performed. Normal distribution was assessed with the Kolmogorov–Smirnov normality test. Normally distributed results were presented as mean ± SD whereas non-normally distributed results were presented as median (25th–75th percentile). The Mann–Whitney test was used to compare groups on continuous variables, and chi-square and Fisher’s exact tests were used to compare categorical variables. Paired *t* test or Wilcoxon’s signed-rank test was performed to compare values at ZEEP and high PEEP, as appropriate. Comparisons of two continuous variables were performed using Spearman’s correlation and linear regression. All comparisons were two-tailed, and *P* < 0.05 was required to exclude the null hypothesis. The areas under the receiver operating characteristic (AUC) curves were compared using a Hanley–McNeil test. The statistical analysis was performed by using the software package SPSS 24.0 (SPSS Inc. Chicago, IL) and MedCalc 11.4.3.0 Software (Mariakerke, Belgium).

## Results

A total of 33 intubated patients were enrolled, and three ARDS patients were excluded due to insufficient respiratory holding time (< 8 s) during the saline injection period for lung perfusion assessment. Twenty-seven out of 30 patients received a high PEEP of 15 cmH_2_O, and 3/30 patients was performed a high PEEP of 12 cmH_2_O during the incremental PEEP trial. Demographics and clinical characteristics are shown in Table 1.

**Table 1:** Demographics and clinical characteristics

Variables	Value
Number of patients	30
Age (years)	54 ± 14
Sex (female/male)	12/18
Weight (kg)	76 ± 24
Patients with high-risk ARDS	10
Post major operation	6/10
Other reasons	4/10
Patients with ARDS	20
Post major operation	11/20
Pneumonia	5/20
Extrapulmonary sepsis	4/20
PaO_2_/FiO_2_ at baseline	242 ± 107
Received vasopressor therapy (%)	23/30
28-day mortality	7/30

*PaO*_*2*_ arterial partial pressure of oxygen, *FiO*_*2*_ fraction of inspiration oxygen

### Effects of PEEP increase

Compared to ZEEP, significantly higher SpO_2_, VQMatch_%_, and EELI were found at high PEEP, whereas mean arterial pressure, DeadSpace_%_, Shunt_%_, and GI were significantly lower (Table 2). Increasing PEEP resulted in recruitment mainly in dorsal regions and overdistension in ventral regions. The median (25th–75th percentile) of O/R ratio was 45% (0.00–112%) and with extremely large variability (range from 0 to 773%). The numbers of patients in the low and high O/R ratio groups were 14 and 16, respectively.

**Table 2:** Comparisons of related parameter between ZEEP and high PEEP in 30 patients

Variables	ZEEP	High PEEP	*P* value
SpO_2_ (%)	95 (91–98)	98 (96–99)	0.008*
HR (bpm)	94 ± 17	94 ± 17	0.783
MAP (mmHg)	87 ± 9	84 ± 10	0.022*
EELI (AU)	1400 (996–1800)	7883 ± 3739	< 0.0001*
GI	0.411 (0.370–0.503)	0.386 (0.350–0.432)	0.131
VQMatch_%_	61 (47–70)	71 ± 10	< 0.0001*
DeadSpace_%_	18 ± 10	13 ± 10	0.013*
Shunt_%_	22 ± 14	16 ± + 11	0.008*
R pixels total	Baseline	142 ± 74	N/A
Ventral	Baseline	32 (13–95)^¶^	N/A
Dorsal	Baseline	82 (53–128)	N/A
O pixels total	Baseline	48 (0–121)	N/A
Ventral	Baseline	48 (0–115)^¶^	N/A
Dorsal	Baseline	0 (0–0.25)	N/A
O/R pixels ratio	Baseline	0.45 (0–1.1)	N/A

*SpO*_*2*_ peripheral capillary oxygen saturation, *HR* heart rate, *MAP* mean arterial pressure, *EELI* end-expiratory lung impedance, *GI* the global inhomogeneity index, *R* recruitment, *O* overdistension, *N/A* not applicable

*Significantly different between ZEEP vs. high PEEP

^¶^*P* < 0.05 compared to dorsal

### Correlation between V-Q matching and recruitment and overdistension

ΔVQMatch_%_ (VQMatch_%_ at high PEEP minus that at ZEEP) was significantly correlated with the numbers of recruited pixels (*r* = 0.468, *P* = 0.009), overdistended pixels (*r* = − 0.666, *P* < 0.001), O/R ratio (*r* = − 0.686, *P* < 0.001), and ΔSpO_2_ (*r* = 0.440, *P* = 0.015).

ΔShunt_%_ (Shunt_%_ at high PEEP minus that at ZEEP) was significantly correlated with the numbers of recruited pixels (*r* = − 0.444, *P* = 0.014), overdistended pixels (*r* = 0.544, *P* = 0.002), and O/R ratio (*r* = 0.580, *P* = 0.001), but not with ΔSpO_2_ (*r* = − 0.355, *P* = 0.055).

ΔDeadSpace_%_ (DeadSpace_%_ at high PEEP minus that at ZEEP) was not correlated with the parameters mentioned above.

### Differences between low O/R ratio and high O/R ratio groups

**Fig. 2:**
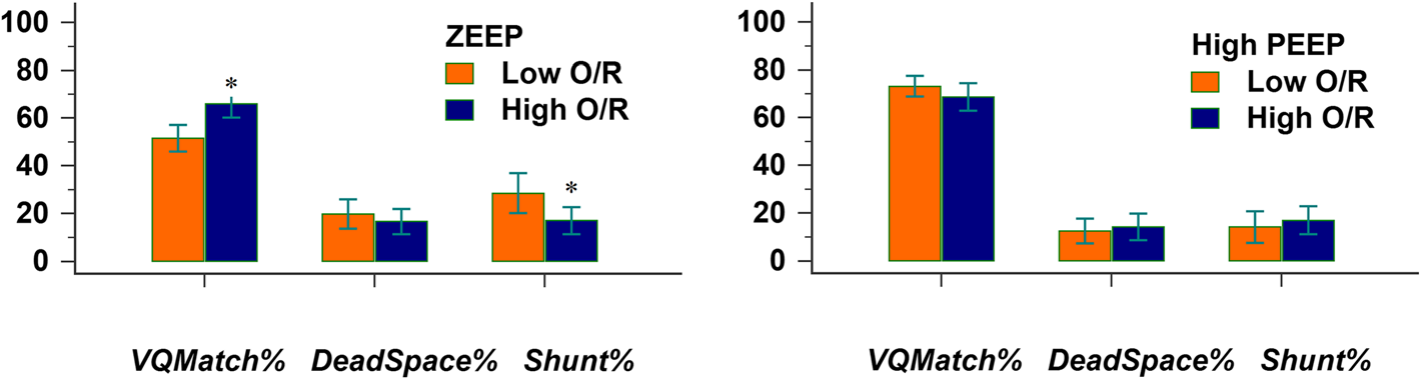
Comparisons of VQMatch_%_, Shunt_%_, and DeadSpace_%_ between low O/R and high O/R groups at ZEEP (left) and PEEP (right). *P < 0.05 compared to low O/R

**Fig. 3:**
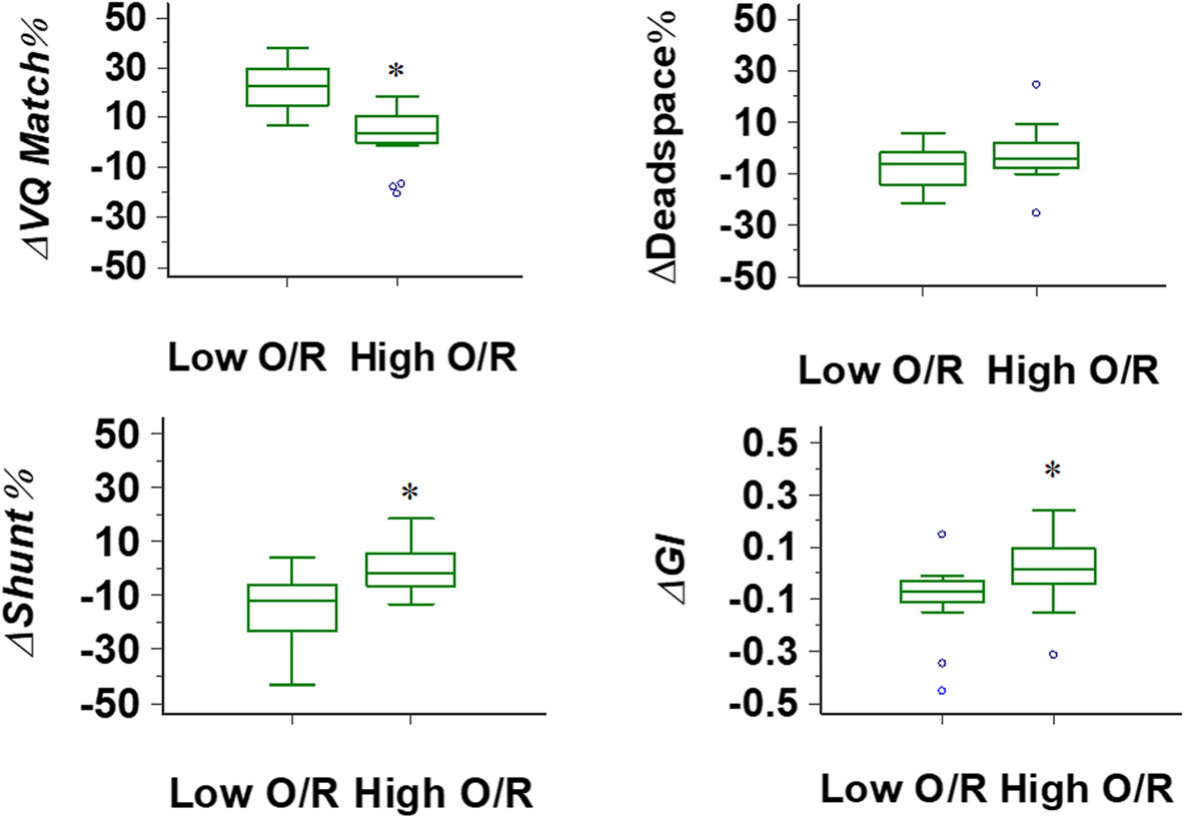
ΔVQMatch_%_, ΔShunt_%_, ΔDeadSpace_%_, and ΔGI induced by PEEP in the low O/R and high O/R groups. Δ *=* high PEEP – ZEEP. *P < 0.05 compared to low O/R

There were no significant differences in PEEP, PaO_2_/FiO_2_, and tidal volume at the baseline between the two groups (Table 3). Patients in the low O/R ratio group (14/30) had significantly higher Shunt_%_ and lower VQMatch_%_ than those in the high O/R ratio group (16/30) at ZEEP but not at high PEEP (Table 3 and Fig. 2). Comparable DeadSpace_%_ was found in both groups. A high PEEP caused a significant improvement of VQMatch_%_, DeadSpace_%_, Shunt_%_, and GI in the low O/R ratio group, but not in the high O/R ratio group (Table 3 and Fig. 3). Diverse responses in VQMatch_%_ (12/16 increase, 4/16 decrease), DeadSpace_%_ (11/16 decrease, 5/16 increase), and Shunt_%_ (10/16 decrease, 6/16 increase) to high PEEP were found in the high O/R ratio group.

**Table 3:** Comparison of the low O/R ratio group and the high O/R group

Variables	Low O/R ratio group *n* = 14	High O/R ratio group *n* = 16	*P* value
Age (years)	57 (45–75)	66 (55–74)	0.275
APACHE II score	16 (14–21)	21 (15–26)	0.166
High-risk ARDS	5/14	5/14	0.703
Mild ARDS	3/14	5/16	0.399
Moderate ARDS	4/14	5/16	1.000
Severe ARDS	2/14	1/16	1.000
FiO_2_ (%)	40 (30–60)	40 (30–50)	0.473
PEEP at baseline (cmH_2_O)	8 (5–10)	6 (5–8)	0.334
PaO_2_/FiO_2_ at baseline	208 (112–355)	233 (187–331)	0.759
Vt at baseline (ml)	410 (400–463)	450 (380–503)	0.759
28-day mortality	3/14	4/16	0.811
HR (bpm)			
ZEEP	91 (81–114)	99 (82–105)	0.951
High PEEP	91 (80–112)	97 (83–105)	0.918
MAP (mmHg)			
ZEEP	86 (82–95)	87 (79–89)	0.886
High PEEP	86 (80–89)	84 (76–97)	0.984
SpO_2_ (%)			
ZEEP	95 (90–99)	97 (91–97)	0.697
High PEEP	99 (96–99)^¶^	97 (94–99)	0.131
ΔSpO_2_ (%)	3 (0–8)	0 (− 1 to 5)	0.093
Shunt_%_			
ZEEP	26 (16–42)	15 (9–24)	0.013*
High PEEP	11 (5–21)^¶^	16 (9–19)	0.580
ΔShunt_%_	− 12 (− 23 to − 5)	− 2 (− 7 to 6)	0.002*
DeadSpace_%_			
ZEEP	20 (11–28)	17 (8–25)	0.448
High PEEP	13 (3–18)^¶^	13 (5–19)	0.580
ΔDeadSpace_%_	− 6 (− 16 to − 1)	− 4 (− 8 to 3)	0.473
VQMatch_%_			
ZEEP	47 (45–61)	68 (59–75)	0.001*
High PEEP	74 (67–80)^¶^	73 (60–78)	0.377
ΔVQMatch_%_	29.7 (22.5–34.4)	3.6 (− 0.3 to 11)	< 0.0001*
GI			
ZEEP	0.427 (0.379–0.504)	0.411 (0.365–0.505)	0.355
High PEEP	0.360 (0.335–0.381)^¶^	0.425 (0.389–0.478)	0.002*
ΔGI	− 0.071 (− 0.121 to − 0.028)	0.015 (− 0.044 to 0.095)	0.015*
EELI (AU)			
ZEEP	1500 (964–1850)	1250 (1000–1775)	0.918
High PEEP	6450 (4400–8325)^¶^	7850 (5450–13,000)^¶^	0.110
ΔEELI (AU)	5000 (3125–6850)	6550 (3650–11,025)	0.093
R pixels			
Total	188 (107–260)	94 (63–145)	0.003*
Ventral	99 (28–146)	22 (4–43)	0.355
Dorsal	102 (36–146)	72 (58–105)	0.001*
O pixels			
Total	0 (0–17)	110 (93–149)	< 0.0001*
Ventral	0 (0–17)	107 (91–147)	< 0.0001*
Dorsal	0 (0–0)	0 (0–4)	0.043*
O/R pixels ratio	0 (0–0.12)	1.0 (0.58–2.20)	< 0.0001*

Δ = high PEEP − ZEEP

*Significantly different comparing the low O/R and high O/R groups

^¶^*R* < 0.05 compared to low PEEP

### Prediction of an increase of VQMatch_%_ > 20% to high PEEP

**Fig. 4:**
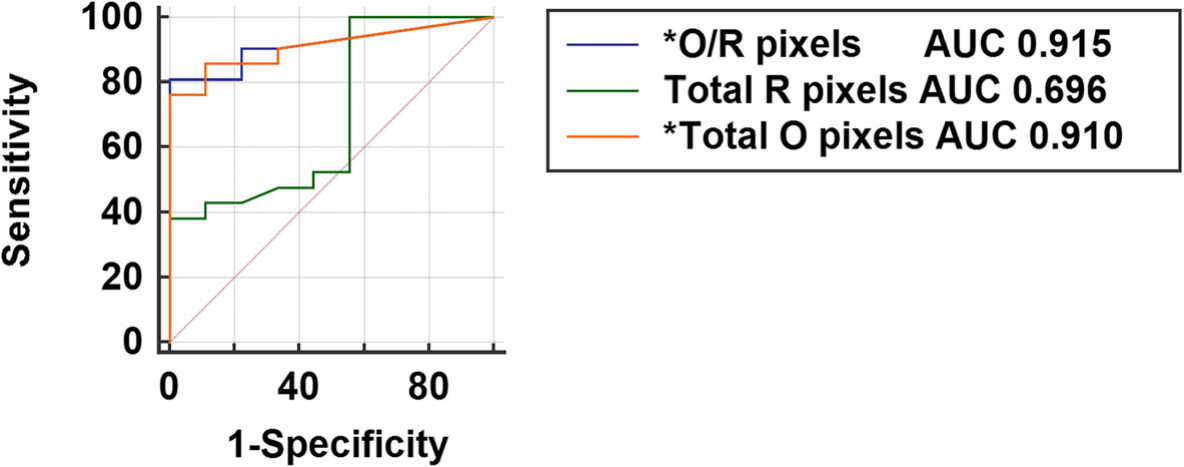
The areas under the receiver operating characteristic curves (AUC) of O/R ratio, total recruited pixels, and total overdistended pixels used for prediction of an increase of VQMatch_%_ > 20% in the incremental PEEP trial. * < 0.05, vs. AUC of total recruited pixels

Ten out of 30 patients had an increase of VQMatch_%_ *>* 20%, and 20/30 patients an increase of VQMatch_%_ < 20% induced by PEEP increase. The AUC of O/R ratio, total recruited pixels, and total overdistended pixels used for prediction of an increase of VQMatch_%_ > 20% to high PEEP are shown in Fig. 4. The O/R ratio has the biggest AUC among the examined parameters. Moreover, both overdistended pixels and O/R ratio have a significantly higher AUC for predicting an increase of VQMatch_%_ to high PEEP than the recruited pixels (*P* < 0.05). Using O/R ratio of 15% resulted in a sensitivity of 81% and a specificity of 100% for an increase of VQMatch_%_ > 20% in response to high PEEP.

## Discussion

In the present study, we found that (1) it was feasible to evaluate the influence of PEEP on lung perfusion combining EIT and hypertonic saline bolus injection. (2) The change of V-Q matching was associated with regional overdistention and recruitment induced by PEEP increase. (3) The benefit of increasing PEEP might be determined by O/R ratio. When O/R < 15%, patients demonstrated more homogeneous ventilation, decrease of dead space and shunt, and increase of V-Q matching and oxygenation. On the contrary, when O/R > 15%, the response to PEEP was rather diverse regarding dead space, shunt, and V-Q matching.

Both shunt and dead space are the determinants of V-Q matching. The primary effect of high PEEP was to improve V-Q match by reducing shunt in ARDS. In theory, for regions with poor alveolar ventilation but sufficient lung blood flow, lung recruitment could decrease the intra-pulmonary shunt. In the present study, the recruited pixels were significantly correlated with ΔShunt_%_ and ΔVQMatch_%_. Karbing et al. recently showed that improvement of lung aeration after PEEP increase was not always consistent with improvement of shunt and V-Q mismatching in 12 ARDS patients [4]. The authors speculated that poorly matched redistribution of V-Q between dependent and non-dependent regions may explain the detrimental changes in shunt and V-Q mismatching after PEEP increase. However, since the V-Q matching was calculated based on global parameters, no regional information can be deduced to prove their hypothesis. With help of EIT and saline bolus injection, our study elegantly showed the relationship between regional V-Q matching and overdistension/recruitment. We found that not only recruitment but also overdistension occurred during PEEP increase. ΔShunt_%_ and ΔVQMatch_%_ were also significantly influenced by the degree of overdistension. The recruited pixels were mainly observed in dorsal (gravity dependent) regions, whereas the overdistended pixels were in ventral (non-gravity dependent) regions. Moreover, we found overdistended pixels had a significantly higher AUC for prediction of an increase of VQMatch_%_ to high PEEP than the recruited pixels (Fig. 4). Hence, more attention should be paid on the regional overdistension induced by high PEEP on V-Q matching.

Simply pursuing maximum lung recruitment without considering the adverse effects of overdistension at high PEEP may worsen the outcomes in mechanically ventilated patients. A recent study found that maximal lung recruitment did not reduce the duration of ventilation-free days or mortality [17]. A parameter of recruitment-to-inflation ratio calculated by systemic pressure-volume curves was proposed to assess lung recruitment and recruited volume during PEEP change from 15 to 5 cmH_2_O [18]. It remains challenging for physicians to balance the regional recruitment and overdistension induced by PEEP. EIT has been used to assess the effect of PEEP on regional recruitment and overdistension in clinical practice [19–23]. Franchineau et al. defined an optimal PEEP would keep regional collapse < 10% with minimum overdistension [20]. Zhao et al. set the PEEP to the cross point of cumulated collapse and overdistension curve [22]. Both studies calculated collapse and overdistension according to the regional compliance curves along PEEP changes. In order to deliver a reliable result, an incremental or decremental PEEP trial with a number of PEEP steps is required [24]. In the current study, we defined recruitment and overdistension based on the ventilation gain and loss, similar to the analysis method introduced previously [25]. Further, we created the O/R ratio to quantify the balance between overdistension and recruitment. An extremely large variability of O/R ratio induced by high PEEP was found in the present study, which indicated diverse responses of lung recruitment and overdistension.

A low O/R ratio indicated lung recruitment with little overdistention. A previous study reported a subgroup of ARDS patients exhibiting recruitment of up to 35% when changing PEEP from 5 to 15 cmH_2_O and with little to no hyperinflation assessed by CT scan at high PEEP [26]. On the contrary, high O/R ratio indicated lung recruitment with high overdistention. When O/R ratio was < 15%, broad beneficial responses were found in homogeneous ventilation, shunt, dead space, and V-Q matching at high PEEP (Table 3). When O/R ratio was > 15%, diverse responses were found in ventilation distribution, shunt, dead space, and V-Q matching. Since the regional overdistension is unavoidable in the high O/R group, selection of high PEEP should be cautiously based on the patient’s condition. It might be difficult to weight risk/benefit of high PEEP in this O/R group, and the prone position might be a good choice. Moreover, we found that using O/R ratio of 15% resulted in a sensitivity of 81% and a specificity of 100% for an increase of VQMatch_%_ > 20% in response to high PEEP. Further study is required to validate whether using O/R ratio to select high PEEP or prone position for ARDS patients could improve the clinical outcome in clinical practice.

Increased dead space fraction is a feature of the early phase of ARDS, and it was associated with the risks of barotrauma and death [27, 28]. The effect of PEEP on dead space is diverse and complicated in ARDS patients. On the one hand, the size of dead space had been used to detect lung collapse and optimize PEEP level after recruitment [29, 30], which was consistent with our current finding that a decrease of dead space fraction could be found in the low overdistension/high recruitment group (low O/R ratio). On the other hand, high PEEP could increase alveolar dead space by increasing the ventilation in overdistended regions without corresponding increase in perfusion. This phenomenon was observed in the high O/R ratio group of the current study. Similarly, Beydon et al. reported a diverse response of dead space to high PEEP in 10 ARDS patients [31]. Gogniat et al. recently reported that Bohr’s dead space could detect different responses to PEEP and individualize lung protective ventilator settings in ARDS patients [32]. Both studies shared the same drawback that no regional dead space could be evaluated. Combining EIT and saline bolus injection, regional V-Q matching provides unique information to understand the profound influence of overdistension and recruitment induced by PEEP.

### Limitations

To our best knowledge, the present study is the first analysis using saline contrast EIT to estimate shunt and dead space related to overdistension and recruitment after PEEP change. Nevertheless, our study must be considered in light of its limitations. (1) Our preliminary study was carried out in a single center with a relatively small number of patients, which reduces the statistical power. (2) The analyzed time intervals at ZEEP and the investigated PEEP steps were relatively short due to ethical reasons, considering the potential influence on patients. Long-term effects of PEEP increase were not evaluated. (3) Cardiac outputs of the patients were not measured. The potential changes in cardiac output induced by PEEP increase might have an impact on V-Q matching, which was not considered in the present study. (4) The thresholds of defining the overdistended and recruited pixels, ventilation and perfusion regions, and change of VQMatch_%_ to high PEEP were rather arbitrary. Further investigations to optimize these thresholds and validate the clinical relevance are warranted. (5) We enrolled patients with ARDS or high-risk ARDS, which might have introduced some heterogeneity. However, studying the effects of higher PEEP on lung recruitment seems clinically relevant for the high-risk ARDS patients, especially in the postoperative patients [33]. (6) The present study should be regarded as a physiologic study of how overdistension/recruitment induced by high PEEP impact V-Q matching since a value of 15 cmH_2_O PEEP was not used in the actual therapy. Further study is required to validate saline contrast EIT method and O/R ratio for an individual mechanical setting and management (such as lung recruitment, PEEP titration, etc.).

## Conclusions

It was feasible to evaluate the influence of PEEP increase on V-Q matching using EIT and hypertonic saline bolus injection. O/R ratio was significantly correlated with V-Q matching. Therefore, this parameter might be able to predict the effects of PEEP on V-Q matching when saline bolus injection is not available, which requires further investigations.

## Data Availability

The datasets used and/or analyzed during the current study are available from the corresponding author on reasonable request.

## Abbreviations

ARDS: Acute respiratory distress syndrome
GI: Global inhomogeneity index
PEEP: Positive end-expiratory pressures
EIT: Electrical impedance tomography
VQMatch: Ventilation–perfusion matching
ZEEP: Zero end-expiratory pressures
O/R: Number of overdistended pixels over the number of recruited pixels
